# Porosity dominates over microgel stiffness for promoting chondrogenesis in zwitterionic granular hydrogels†

**DOI:** 10.1039/d4bm00233d

**Published:** 2024-09-30

**Authors:** Maryam Asadikorayem, Lucia G. Brunel, Patrick Weber, Sarah C. Heilshorn, Marcy Zenobi-Wong

**Affiliations:** a Department of Health Sciences and Technology ETH Zürich Zürich Switzerland marcy.zenobi@hest.ethz.ch; b Department of Chemical Engineering, Stanford University Stanford CA USA; c Department of Materials Science and Engineering, Stanford University Stanford CA USA

## Abstract

Granular hydrogels comprised of jammed, crosslinked microgels offer great potential as biomaterial scaffolds for cell-based therapies, including for cartilage tissue regeneration. As stiffness and porosity of hydrogels affect the phenotype of encapsulated cells and the extent of tissue regeneration, the design of tunable granular hydrogels to control and optimize these parameters is highly desirable. We hypothesized that chondrogenesis could be modulated using a granular hydrogel platform based on biocompatible, zwitterionic materials with independent intra- and inter-microgel crosslinking mechanisms. Microgels are made with mechanical fragmentation of photocrosslinked zwitterionic carboxybetaine acrylamide (CBAA) and sulfobetaine methacrylate (SBMA) hydrogels, and secondarily crosslinked in the presence of cells using horseradish peroxide (HRP) to produce cell-laden granular hydrogels. We varied the intra-microgel crosslinking density to produce microgels with varied stiffnesses (1–3 kPa) and swelling properties. These microgels, when resuspended at the same weight fraction and secondarily crosslinked, resulted in granular hydrogels with distinct porosities (5–40%) due to differing swelling properties. The greatest extent of chondrogenesis was achieved in scaffolds with the highest microgel stiffness and highest porosity. However, when scaffold porosity was kept constant and just microgel stiffness varied, cell phenotype and chondrogenesis were similar across scaffolds. These results indicate the dominant role of granular scaffold porosity on chondrogenesis, whereas microgel stiffness appears to play a relatively minor role. These observations are in contrast to cells encapsulated within conventional bulk hydrogels, where stiffness has been shown to significantly affect chondrocyte response. In summary, we introduce chemically-defined, zwitterionic biomaterials to fabricate versatile granular hydrogels allowing for tunable scaffold porosity and microgel stiffness to study and influence chondrogenesis.

## Abstract 2

A zwitterionic granular hydrogel with tunable porosity and microgel stiffness is used for cartilage tissue engineering. This chemically-defined biomaterial provides a versatile platform for investigation and optimization of scaffold parameters.

## Graphical abstract

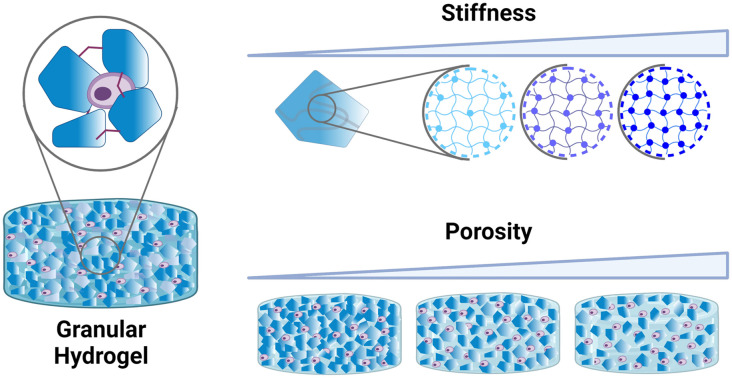

## 1. Introduction

Approximately 350 million patients worldwide suffer from cartilage lesions caused by injury, disease, or wear-and-tear, significantly affecting their mobility and quality of life.^1^ Cartilage damage is becoming increasingly prevalent due to the aging global population and increased life expectancy.^2^ Unfortunately, cartilage tissue has low capacity for self-regeneration.^3^ Developing treatments that not only alleviate the symptoms of cartilage degeneration but also restore function to the tissue is therefore of great clinical consequence and importance.^4^ Autografts and allografts are common therapies for repairing small cartilage lesions but suffer from insufficient availability and quality of the donor tissue as well as reduced contour matching and load-bearing capacities.^5–7^ Over the past few decades, tissue engineering strategies consisting of cells, scaffolds, and bioactive factors have emerged to improve the functionality of damaged or diseased cartilage.^8–10^ The delivery of living chondrocytes—the primary cell type in cartilage—to a cartilage defect has clinically demonstrated enhanced chondrogenesis.^11–14^ To protect cells during injection, retain their spatial localization within the body, and provide a suitable microenvironment for cell survival and tissue regeneration after implantation, hydrogel biomaterials have been leveraged as bioengineered cell carriers.^15^

Hydrogels are water-swollen polymeric materials that provide three-dimensional (3D) environments that can be tuned to recapitulate biochemical and biophysical features of the native extracellular matrix (ECM). However, conventional bulk hydrogels are nanoporous, often limiting the rate of nutrient and metabolite diffusion and cell infiltration through the material.^16^ Over the past decade, the fabrication of granular hydrogels has emerged as a facile strategy to create microporous scaffolds for tissue engineering applications.^17,18^ The structural units of a granular hydrogel are microgels, which can be jammed together into an injectable, dynamic material with shear-thinning and self-healing properties.^19,20^ The jammed microgels are then crosslinked to each other to form a stable scaffold with intrinsic microporosity, increased mass transport and cell migration.^21^ When the microgels are crosslinked together, the scaffolds are often termed “Microporous Annealed Particle (MAP)” scaffolds, due to their stably-linked interconnected microporosity resulting from the microgel building blocks.^17,22^ These granular hydrogels have shown potential for advancing biomedical technologies as customizable platforms for a wide range of regenerative medicine applications.^21^ For cartilage tissue engineering, improved chondrogenesis has been demonstrated both *in vitro* and *in vivo* for chondrocytes and mesenchymal stromal cells in gelatin microgels compared to bulk gelatin hydrogels.^23–25^ Furthermore, in an *ex vivo* cartilage explant culture, acellular hyaluronic acid granular hydrogels facilitated increased chondrocyte migration from native cartilage tissue and enhanced matrix deposition compared to bulk hydrogels.^26^

Many types of hydrogel materials have been applied to cartilage tissue engineering, ranging from natural biopolymers such collagen, fibrin, and hyaluronic acid, to synthetic polymers such as polylactic acid, poly-glycolic acid, poly(ε-caprolactone).^27–29^ The use of zwitterionic materials in tissue engineering has proven especially attractive for bioengineered tissues intended for transplantation due to their excellent biocompatibility, low immunogenicity, and high resistance to fouling.^30–32^ Zwitterionic polymers contain equal numbers of cationic and anionic groups in close proximity within their repeating unit, imparting an overall neutral charge and a strong hydration effect.^33^ Hydrogels composed of zwitterionic polymers demonstrate increased resistance against foreign body reaction, minimizing inflammatory responses during the material's implantation and degradation.^34–36^ We recently described an approach for fabricating zwitterionic granular hydrogels by mechanical fragmentation that are injectable and allow for direct chondrocyte encapsulation and ECM production.^37^ In this system, granular hydrogels were formed from bulk hydrogels made of the zwitterionic comonomers carboxybetaine acrylamide (CBAA) and sulfobetaine methacrylate (SBMA), which were photocrosslinked together with gelatin methacryloyl (GelMA). All microgels were formed from the same bulk hydrogels and therefore had the same stiffness. By increasing the size of the microgels, the void fraction of the granular hydrogel scaffold increased from ∼13% to ∼20%, resulting in enhanced proliferation and chondrogenesis of the encapsulated chondrocytes.^37^ Moreover, we recently showed that these zwitterionic granular hydrogels exhibit low immunogenicity and anti-fibrotic properties when implanted in immunocompetent animal models.^38^

The unique properties of microgels—such as their size, shape, and composition—are critical in influencing the overall characteristics of granular hydrogel scaffolds.^39^ For example, the size and shape of microgels have commonly been modulated through the fabrication strategy (*e.g.*, batch or microfluidic emulsions, lithography, electrohydrodynamic spraying, or mechanical fragmentation) to change the porosity of granular hydrogel scaffolds.^22,39^ In turn, the granular hydrogel properties may affect the behavior of encapsulated cells, such as their migration, proliferation, matrix deposition, and integration with the native tissue after implantation.^17,40,41^ Hydrogel stiffness and porosity have been particularly shown to be critical factors affecting chondrogenesis of encapsulated cells.^26,37,42–48^ However, these two parameters are often intertwined and therefore challenging to decouple in both bulk and granular hydrogels. In bulk hydrogels, increased stiffness is often achieved through increased crosslinking density, which in turn decreases the hydrogel pore or mesh size. Granular scaffolds, in which cells can be encapsulated in pores between microgels, can therefore serve as a versatile platform to study the effect of independently varying the microporosity and microgel stiffness. This requires a careful design of the granular scaffold properties and independent intra-microgel and inter-microgel crosslinking strategies. In one study on cells encapsulated within granular hydrogel scaffolds, using microgels with increasing crosslinking densities and thus increasing stiffnesses (∼200 Pa to ∼1000 Pa) resulted in increased spreading of fibroblasts.^40^ However, in this study, the change in crosslinking density affected not only the porosity but also the conjugated RGD ligand presentation, creating a confounding variable.

**Fig. 1:**
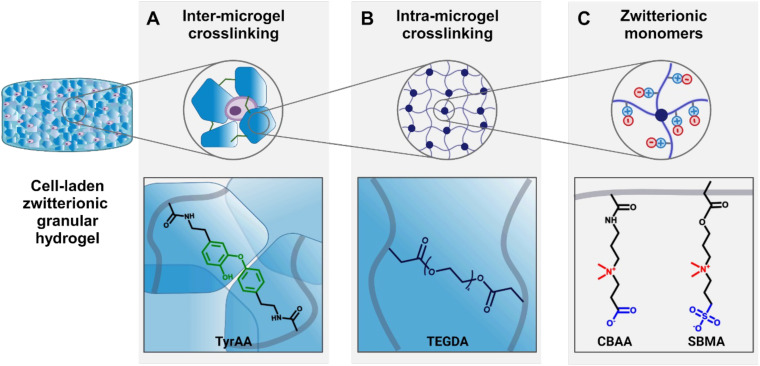
Schematic representation of the tunable zwitterionic granular hydrogel. (A) Enzymatic inter-microgel crosslinking occurs by covalent bond formation between tyramine acrylamide (TyrAA) moieties within microgels. (B) Intra-microgel crosslinking occurs by photocrosslinking of the zwitterionic monomers using tetra(ethylene glycol) diacrylate (TEGDA) crosslinker. (C) Carboxybetaine acrylamide (CBAA) and sulfobetaine methacrylate (SBMA) are used as zwitterionic monomers for hydrogel preparation.

In this current work, we aimed to explore how the properties of zwitterionic granular hydrogels affect chondrogenesis by leveraging solely the degree of crosslinking of the bulk hydrogel to modulate both the microgel stiffness and the scaffold porosity. We adopted a synthetic small molecule crosslinker, tetra(ethylene glycol) diacrylate (TEGDA), to create a versatile, fully chemically-defined, tunable material system of zwitterionic CBAA and SMBA hydrogels. Moreover, we used an enzymatic tyramine-based crosslinking as an independent inter-microgel crosslinking mechanism to form the granular hydrogels (Fig. 1).

First, we demonstrated that by controlling the crosslinking density of the bulk hydrogels from which microgels are made, we could tune both the stiffness of the microgels (1–3 kPa) and the porosity of the scaffold (5–40%) across a wide range. The microgel size and weight fraction within the scaffold are kept constant, therefore decoupling porosity from the size and shape of the microgels, as opposed to conventional approaches.^22,39^ Then, we demonstrated that by changing the weight fraction at which the microgels were resuspended to account for their differing equilibrium water contents, scaffolds had identical porosities but different microgel stiffnesses. Finally, using these zwitterionic granular hydrogels as a material platform, we studied the effects of the crosslinking density on the microgel stiffness and the granular hydrogel scaffold porosity, as well as the resultant effects on the behavior of encapsulated chondrocytes *in vitro*. In summary, this research establishes a foundational material platform and design insights to interrogate the impact of key granular hydrogel material properties on the maturation of engineered tissues. We specifically elucidate the chondrogenesis process within zwitterionic granular hydrogels, thereby informing the design of improved bioengineered therapies for cartilage regeneration.

## 2. Experimental section

### 2.1. Materials

Beta-propiolactone and *N*,*N*-dimethylformamide (DMF) were purchased from Acros. *N*-(3-(Dimethylamino)propyl)acrylamide (DMAPA) and l-ascorbic acid 2-phosphate Sesquimagnesium salt hydrate were purchased from TCI chemicals. Gentamycin, Dulbecco's modified Eagle's medium (DMEM), fetal bovine serum (FBS), and Antibiotic–Antimycotic (Anti–Anti) were purchased from Gibco. ITS+ Premix Universal Culture Supplement was purchased from Corning. Fibroblast growth factor-2 (FGF-2) and transforming growth factor-β3 (TGF-β3) were purchased from PreproTech. All other reagents and solvents were purchased from Sigma unless indicated otherwise.

### 2.2. Monomer and crosslinker syntheses

#### 2.2.1. Carboxybetaine acrylamide (CBAA) synthesis

The CBAA monomer was synthesized as previously described^49^ with minor modifications. In a 100 mL round-bottom flask with a magnetic stir bar, DMAPA (8.9 g, 57.28 mmol, 1 eq.) was dissolved in 60 mL anhydrous tetrahydrofuran (THF). The flask was sealed with a dropping funnel and placed in a −10 °C ethanol bath. Beta-propiolactone (5 mL, 79.86 mmol, 1.4 eq.) dissolved in 15 mL anhydrous THF was added to the DMAPA solution dropwise while stirring for 2 h. The reaction mixture was then allowed to warm to room temperature and stirred overnight. The resulting white suspension was chilled at −20 °C for 24 h to precipitate the product. The mixture was then filtered through a sintered glass funnel (S4 porosity) and washed with dry diethyl ether. The product was obtained with vacuum filtration, washed several times with cold ether, and dried overnight under high vacuum. ^1^H NMR (Bruker Ultrashield 400 MHz) in deuterated water (D_2_O) was used to confirm successful and pure synthesis of the product.

#### 2.2.2. Tyramine acrylamide (TyrAA) synthesis

The TyrAA monomer was synthesized as previously described^50^ with minor modifications. In a 100 mL round-bottom flask with a magnetic stir bar, tyramine hydrochloride (2.0 g, 11.52 mmol, 1 eq.) was dissolved in 32 mL DMF, and *N*,*N*-diisopropylethylamine (DIPEA) (6 mL, 34.55 mmol, 3 eq.) was added. The solution was degassed by bubbling with nitrogen gas for 15 min and cooled in an ice bath. Acryloyl chloride (1.2 mL, 14.97 mmol, 1.3 eq.) dissolved in 3 mL DMF was slowly added dropwise while vigorously stirring. The reaction mixture was then allowed to warm to room temperature and stirred overnight. The solvent was then removed using a rotary evaporator. The product was redissolved in 10 mL ethyl acetate and chilled at −20 °C overnight to allow for product crystallization. The product was obtained with vacuum filtration, washed with cold chloroform, and dried overnight under high vacuum. ^1^H NMR (Bruker Ultrashield 400 MHz) in D_2_O was used to confirm successful and pure synthesis of the product.

#### 2.2.3. Gelatin methacrylate (GelMA) synthesis

GelMA was synthesized through the modification of gelatin type A as previously described.^51^ The degree of substitution (DS) was determined with ^1^H NMR (Bruker Ultrashield 400 MHz) in D_2_O. The GelMA lysine integration signal (2.95–3.05 ppm) was compared to the unmodified gelatin lysine integration signal (2.95–3.05 ppm). The phenylalanine signal (7.2–7.5 ppm) was used as an internal reference. The GelMA DS was found to be approximately 73%.

### 2.3. Zwitterionic granular hydrogel preparation and characterization

#### 2.3.1. Bulk hydrogels

Bulk zwitterionic hydrogels were produced by photopolymerizing the zwitterionic monomers and TyrAA using TEGDA or GelMA as the crosslinker and LAP as the photoinitiator. The solution was prepared by dissolving CBAA, SBMA, and TyrAA in Milli-Q water at 1.875 M, 0.625 M (25 mol% of CBAA), and 0.125 M (5 mol% of CBAA) final concentrations, respectively. TyrAA was dissolved first at 50 wt% in DMF before its addition to the solution. TEGDA was added to the solution to have a final concentration of 0.0025 M, 0.005 M, and 0.0125 M for LOW, MEDIUM and HIGH concentrations respectively, such that there were 1, 2, and 5 mmol of TEGDA per 1 mol of zwitterionic monomers. When GelMA was used as a crosslinker instead of TEGDA, the final concentration of GelMA was adjusted to have 2 mmol of methacryloyl groups per 1 mol of zwitterionic monomers. To prepare fluorescently labeled hydrogels, acryloxyethyl thiocarbamoyl rhodamine B comonomer (0.015 wt%) was also added to the starting solution. Immediately prior to photocrosslinking, LAP (0.05 wt%) was mixed into the solution. The solution was injected between two glass slides with a 1 mm polytetrafluoroethylene spacer. Photopolymerization was initiated by UV-VIS (405 nm, 15 min), and the resulting hydrogels were dialyzed against deionized (DI) water for 5 days with daily water changes. At the end of dialysis, hydrogel disks (6 mm diameter) were punched out, weighed, and freeze-dried to measure the equilibrium water content (EWC) of the hydrogels. EWC was measured as the ratio of water mass (swollen hydrogel weight minus dried hydrogel weight) to swollen hydrogel mass. For mechanical testing of the bulk hydrogels, unconfined compression experiments were performed on a TA.XTplus texture Analyzer (Anton Paar) equipped with a 500 g load cell. For each sample, a pre-load was applied to the sample until it reached full contact with the plate and was then allowed to relax completely. Samples were compressed at a rate of 0.01 mm s^−1^ until they reached 15% strain. The compressive modulus was extracted from the slope of the first linear part of the stress *vs*. strain curve.

#### 2.3.2. Microgels

Zwitterionic microgels were fabricated through the mechanical fragmentation of equilibrated bulk zwitterionic hydrogels. The bulk hydrogels were cut into small pieces and transferred into a 10 mL custom-made extruder. They were manually extruded three times through a metal sieve with a mesh size of 90 μm. Microgels were then sterilized in 70% ethanol, dried overnight in the vacuum oven, re-suspended in sterile water, and lyophilized. To determine the microgel size distribution, fluorescently labeled zwitterionic microgels were resuspended in phosphate-buffered saline (PBS) at a low concentration of ∼0.5% to permit microgel separation. The microgel sizes were determined by dispersing the microgels between glass slides and imaging them with an AxioObserver inverted epifluorescence microscope (AptoTome.2, ZEISS). Image analysis was performed with FIJI (ImageJ2, version 2.3.0/1.53f)^52^ by setting the threshold to select for the microgels and running particle analysis.

#### 2.3.3. Granular hydrogel scaffolds

To make zwitterionic granular hydrogel scaffolds, the lyophilized microgels were resuspended at 3–8 wt% in PBS. To prepare 100 μL of the granular hydrogel scaffold, 90 μL of microgels were mixed with 5 μL of horseradish peroxidase (HRP, 2 mg mL^−1^, 300 U mL^−1^) and 5 μL of hydrogen peroxide (H_2_O_2_, 0.1%), cast in polydimethylsiloxane (PDMS) molds, and incubated for 30 min. To measure the porosity of granular hydrogel scaffolds, fluorescently labelled microgels were prepared and crosslinked to obtain fluorescent granular hydrogel scaffolds. Samples were imaged using confocal microscopy (SP8, Leica). Porosity was determined with FIJI (ImageJ2, version 2.3.0/1.53f)^52^ by setting the threshold to select for the void spaces of single images within *z*-stacks. The fraction occupied by voids was determined for each image and averaged for the whole stack. Compression tests were performed identically as described for the bulk hydrogels.

### 2.4. *In vitro* chondrogenesis

#### 2.4.1. Primary human chondrocyte isolation and culture

Primary human chondrocytes were collected from corrective surgeries of polydactyly patients as described previously.^53^ Experiments were performed in accordance with the guidelines of “Ordinance on human research with the exception of clinical trials (from the Swiss Federal Council)”, and approved by the Canton of Zürich Ethics Commission (license number PB_2017-00510/2022-01455). Informed consents were obtained from legal guardians of participants of this study. Cartilage pieces were finely sliced (∼0.5 mm thickness), washed extensively in PBS with 50 μg mL^−1^ gentamicin, and digested in collagenase solution (DMEM, 1000 CDU mL^−1^ collagenase from Clostridium histolyticum, 2 V% FBS, 1× Anti–Anti) overnight with gentle shaking at 37 °C. The resulting cell suspension was passed through a 40 μm cell strainer, and the cell pellet was collected by centrifugation (500 rcf, 10 minutes). The cells were plated at 10 000 cells per cm^2^ and expanded in DMEM, 10 V% FBS, 1× Anti–Anti and 10 ng mL^−1^ FGF-2 at 37 °C, 5% CO_2_ and 95% humidity. After the first passage, the seeding density was reduced to 3000 cells per cm^2^ and Anti–Anti was exchanged for 10 μg mL^−1^ gentamicin.

#### 2.4.2. Chondrocyte encapsulation in zwitterionic granular hydrogel scaffolds

Primary human chondrocytes at passage 3 were trypsinized and mixed with zwitterionic microgels at a final density of 10 million cells per mL. The microgel-cell suspension was then crosslinked as described previously, in cylindrical PDMS molds with 4 mm diameter and 2 mm height. Scaffolds were cultured in chondrogenic medium containing DMEM, 10 μg mL^−1^ gentamycin, 1% ITS+, 50 μg mL^−1^l-ascorbate-2-phosphate, 40 μg mL^−1^l-proline, and 10 ng mL^−1^ TGF-β3. The medium was replaced every other day.

#### 2.4.3. Cell spreading and viability

To assess viability, samples were stained with a medium supplemented with 1 μm Calcein AM, 1 μm propidium iodide (PI), and 0.3 μM Hoechst for 1 h. For samples with fluorescent microgels, PI was not used. Imaging was performed on a Leica SP8 confocal microscope equipped with a 10× objective. *Z*-stacks were acquired with a height of 100 μm and step size of 10 μm. Viability was assessed by counting viable (Calcein AM+) and dead (PI+) cells and dividing the number of viable cells by the total number of viable cells plus dead cells. Cell spread area was determined by using a threshold to select the Calcein AM+ stain and measuring the fraction of cell coverage over the imaged area. All image analysis was performed with FIJI (ImageJ2, version 2.3.0/1.53f).^52^

#### 2.4.4. Histology and immunohistochemistry

Samples were fixed in 4% paraformaldehyde for 4 h, dehydrated in an ethanol sequence, embedded in paraffin wax (Milestone LogosJ) and sectioned into 5 μm slices on a microtome. Samples were progressively deparaffinized and rehydrated before staining. *Safranin O staining*: Sections were stained in Weigert's Iron Hematoxylin solution for 5 min, washed in DI water, and differentiated in 1% acid–alcohol for 2 s. Sections were then washed again, stained in 0.02% Fast Green solution for 1 min, and rinsed with 1% acetic acid for 30 s. Finally, sections were stained in 1% Safranin O for 30 min, dehydrated to xylene, and mounted. *Collagen I and II immunohistochemistry*: Antigen retrieval was performed in hyaluronidase (1200 U mL^−1^) at 37 °C for 30 min. Sections were blocked with 5% BSA in PBS for 1 h. The primary antibodies—rabbit anti-collagen I (1 : 1500, ab138492, Abcam) and mouse anti-collagen II (1 : 20, II-II6B3-s, DSHB Hybridoma)—were dissolved each in 1% BSA in PBS, and sections were incubated in the primary antibody solution overnight at 4 °C. Sections were then incubated with the secondary antibody—goat anti-rabbit IgG–HRP for collagen I (1 : 1000, ab6721, Abcam), or goat anti-mouse IgG–HRP for collagen II (1 : 1000, ab6789, Abcam)—in 1% BSA in PBS for 1 h and developed with the DAB substrate kit (ab64238, Abcam) for 5 min. Sections were stained with Weigert's iron hematoxylin (Thermo Fisher Scientific) for 3 min, destained in 1% acid–alcohol, blued in 0.1% Na_2_CO_3_, dehydrated to xylene, and mounted. Brightfield images of all stained sections were recorded on a 3DHistech Pannoramic 250-slide scanner and visualized with CaseViewer 2.4. Semi-quantitative analyses of glycosaminoglycans (GAG), collagen I, and collagen II were performed following an established protocol.^54^ First, colour deconvolution was performed to isolate the DAB and Safranin O from Hematoxylin in collagens and Safranin O stainings, respectively. Then, a threshold was set to isolate secreted matrix from microgels, and the images were analysed by measuring the mean grey value. All images were analysed in the same way and normalized to the average mean grey value measured for HIGH group in each experiment.

### 2.5. Statistical analysis

Statistical analyses were performed using GraphPad Prism (version 9.5). All data are presented as mean ± standard deviation with *n* ≥ 3 unless indicated otherwise. Normality of data sets was assessed with the Shapiro–Wilk test. Comparisons of hydrogel equilibrium water content, microgel size, porosity, cell spreading area, and cell viability were assessed with a one-way analysis of variance (ANOVA) followed by Tukey's multiple comparisons *post-hoc* test. Comparisons of elastic modulus were assessed with an unpaired *t*-test for populations with the same standard deviations or unpaired *t*-test with Welch's correction for populations with unequal standard deviations. A level of *p* < 0.05 was considered significant. For all data comparisons, the *p*-values for statistical significance are represented as follows: * *p* < 0.05, ** *p* < 0.01, *** *p* < 0.001, **** *p* < 0.0001, and ns = not significant.

## 3. Results and discussion

### 3.1. Fabrication of chemically-defined zwitterionic granular hydrogels with tunable microgel stiffness and scaffold porosity

While the transplantation of cell-based therapies for cartilage tissue engineering holds great promise, the inflamed environment in a cartilage defect can be exacerbated by immunogenicity of the cell-laden biomaterial.^55^ The inflammatory agents released during the activation of immune pathways impair the cartilage healing process.^56^ Zwitterionic materials, therefore, are attractive for their high cytocompatibility and low immunogenicity, evading detection from the immune system.^57^ In this work, we define a system of zwitterionic granular hydrogels that support viability and chondrogenesis of encapsulated chondrocytes. We show that by modulating the extent of hydrogel crosslinking, the microgel stiffness and scaffold porosity can be tuned. The hydrogels are made from the zwitterionic monomers CBAA and SBMA. CBAA is highly anti-fouling due to its high degree of hydration.^58^ SMBA is also anti-fouling^59^ but supports greater cell attachment than other zwitterionic monomers,^60^ likely due to the presence of anionic SO_3_^−^ groups.^61^ We have previously demonstrated that these zwitterionic monomers can be photocrosslinked using GelMA as a crosslinker to create bulk hydrogels.^37^ As GelMA is a form of gelatin, it is a naturally-derived biopolymer that contains cell-adhesive ligands, but it is not chemically-defined and can suffer from batch-to-batch variation. In this work, we introduce TEGDA as a synthetic, small molecule crosslinker for a fully chemically-defined zwitterionic material platform with tunable properties. The bulk hydrogels are formed by photocrosslinking a solution of CBAA, SMBA, and TyrAA monomers with TEGDA as the crosslinker (ESI Table 1†). The bulk hydrogels are mechanically fragmented into microgels, which are then crosslinked together to form a granular scaffold through enzymatic crosslinking of the tyramine moieties on the TyrAA in the hydrogels. The TyrAA comonomer provides phenol groups which can be covalently crosslinked in the presence of HRP and H_2_O_2_, allowing for fast and stable annealing of microgels.

**Fig. 2:**
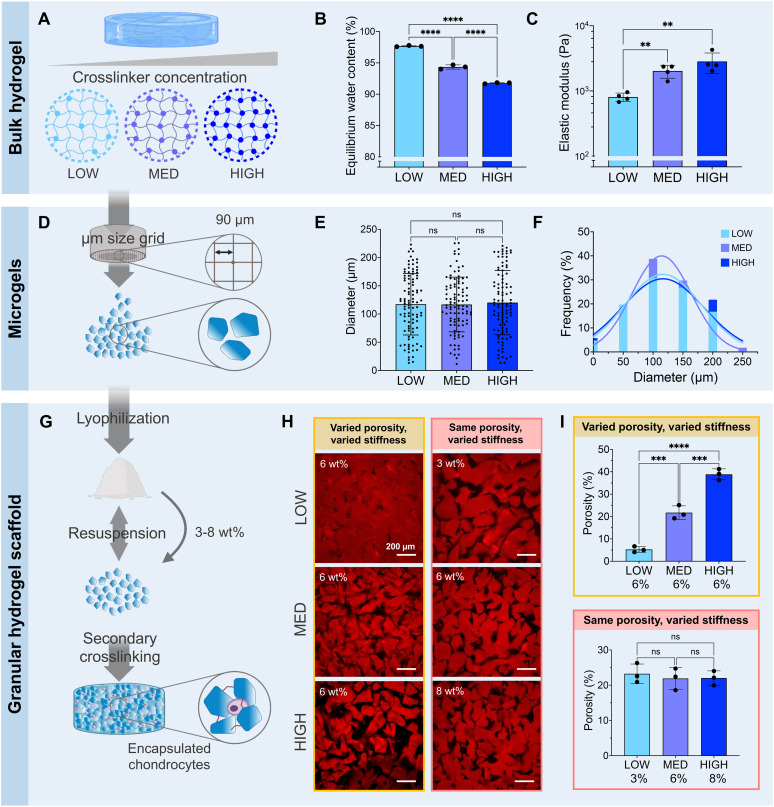
Fabrication and characterization of zwitterionic hydrogel scaffolds. (A) Bulk hydrogels are photocrosslinked with varying concentrations of the TEGDA crosslinker. LOW = 1 mmol, MED = 2 mmol, HIGH = 5 mmol TEGDA. (B) The equilibrium water content decreases and (C) the elastic modulus of the bulk hydrogel increases with increasing TEGDA crosslinker concentration. (D) Microgels are fabricated by mechanically fragmenting the photocrosslinked bulk hydrogels using a μm-sized grid. (E) The average microgel diameter and (F) the distributions of microgel diameters are similar for microgels made from bulk hydrogels with LOW, MED, or HIGH concentrations of the TEGDA crosslinker. (G) The microgels are lyophilized and then resuspended in PBS prior to use. Encapsulation of chondrocytes between the microgels and secondary crosslinking of the microgels results in a microporous, cell-laden granular hydrogel scaffold. (H) Representative fluorescence images and (I) porosity quantification of granular hydrogel scaffolds made with LOW, MED, or HIGH concentrations of the TEGDA crosslinker. Microgels were either resuspended all at 6 wt% to create scaffolds with a varied porosity and varied microgel stiffness, or resuspended at the concentration that allows for the same porosity across all conditions to create scaffolds with the same porosity but varied microgel stiffness.

Granular hydrogel scaffolds were fabricated from engineered zwitterionic materials to have tunable properties (Fig. 2). First, bulk hydrogels made with CBAA and SBMA zwitterionic comonomers were crosslinked with a LOW, MED, or HIGH concentration of the TEGDA crosslinker, corresponding to 1, 2, and 5 mmol of TEGDA per 1 mol of zwitterionic monomers (Fig. 2A). The bulk zwitterionic hydrogels were dialyzed and swollen to equilibrium in water. As expected, the swelling potential and consequently the equilibrium water content decreased with increasing amounts of crosslinker, with 97, 94, and 92 wt% water for LOW, MED, and HIGH TEGDA concentrations, respectively (Fig. 2B). The different extent of crosslinking also affected the elastic modulus of the bulk hydrogels. The elastic modulus increased from 800, 2000, and 3000 Pa for bulk hydrogels with LOW, MED, and HIGH TEGDA concentrations, respectively (Fig. 2C). Hydrogels that are more densely crosslinked have smaller polymer mesh sizes, decreasing their water uptake capacity and thus resulting in stiffer materials.^62^

All bulk hydrogels were mechanically fragmented into microgels by manually pressing them through custom-built extruders (Fig. 2D). The mechanical fragmentation technique was chosen due to its simplicity, scalability, and cost-effectiveness. The extruders were fitted with a metal sieve with a grid size of 90 μm. For bulk gels with LOW, MED, and HIGH TEDGA concentrations, the resultant microgels all had average diameters of 120 μm and similar particle size distributions (Fig. 2E and F). While microgels made from mechanical fragmentation have irregular shapes (Fig. S1†) and a wider particle size distribution than spherical microgels fabricated with microfluidic devices or batch emulsions, the heterogeneous and polygonal nature of the constituent microgels allows the resultant granular hydrogel scaffolds to reach overall higher stiffnesses and number of pores.^39^ Since the LOW, MED, and HIGH TEGDA microgels were made with different amounts of TEDGA crosslinker but with the same extruder mesh size for mechanical fragmentation, the microgels had the same average size but different stiffnesses.

The zwitterionic microgels with LOW, MED, and HIGH TEGDA were sterilized and lyophilized for storage. Before experimental use, the microgels were resuspended at the desired concentration by hydrating the lyophilized particles in PBS. For studies with encapsulated chondrocytes, the cells were mixed into the microgel slurry. A secondary crosslinking step was then initiated by adding HRP and H_2_O_2_ to enzymatically crosslink the microgels together. Covalent bonds are created between the phenol groups of the TyrAA comonomer that was incorporated into the bulk hydrogels to achieve inter-microgel crosslinking and form a granular hydrogel scaffold (Fig. 2G). When the microgels with LOW, MED, and HIGH TEGDA were rehydrated each at 6 wt% in PBS (“wt% matched”), the resultant granular scaffolds had notably different porosities, with porosity increasing for microgels made with increasing amounts of the TEGDA crosslinker (Fig. 2H, left). Since hydrogels with a lower degree of crosslinking have a higher equilibrium water content, they are less polymer dense. Therefore, resuspending the lyophilized microgel particles at the same weight fraction means that more microgels of the less polymer dense material (*e.g.*, LOW TEGDA hydrogels) will be incorporated into the granular hydrogel scaffold compared to microgels of the more polymer dense material (*e.g.*, HIGH TEGDA hydrogels). Porosities of the LOW, MED, and HIGH TEGDA granular scaffolds were 5, 20, and 40%, respectively (Fig. 2I, top). Interestingly, this demonstrates modulation of the porosity without changing the microgel size, a common way of varying void fraction within a granular hydrogel scaffold.^63,64^ Furthermore, while the reported porosity of scaffolds made from mechanically fragmented microgels have typically ranged between 2–20%,^39,47^ here we demonstrate crosslinked scaffolds of mechanically fragmented microgels with up to 40% porosity.

Microgels with LOW, MED, and HIGH TEGDA could also be resuspended at different weight fractions to account for their different equilibrium water contents, such that the porosities of the scaffolds were matched (Fig. 2H, right). The LOW TEGDA microgels (EWC ∼ 97 wt%) were resuspended at 3 wt%, the MED TEGDA microgels (EWC ∼ 94 wt%) were resuspended at 6 wt%, and the HIGH TEGDA microgels (EWC ∼ 92 wt%) were resuspended at 8 wt%. Under these conditions, the porosities of the LOW, MED, and HIGH TEGDA granular scaffolds were all in the range of 20% (Fig. 2I, bottom). Thus, zwitterionic hydrogel scaffolds were fabricated from microgels of the same size but different crosslinker concentrations, allowing tunability over the microgel stiffness and the scaffold porosity.

As TEGDA is a synthetic small molecule crosslinker, the zwitterionic granular hydrogel system described here is fully chemically-defined while still offering similar hydrogel crosslinking behavior as GelMA (Fig. S2†). For the same amount of crosslinker (2 mmol of methacryloyl groups in GelMA or 2 mmol of the TEGDA crosslinker per 1 mol of zwitterionic monomers), zwitterionic bulk gels were formed with similar equilibrium water contents and elastic moduli (Fig. S2A†). After mechanical fragmentation of the bulk gels into microgels (Fig. S2B†) and secondary crosslinking of the microgels into a granular scaffold, the scaffolds made with both crosslinkers were stable over 4 weeks in aqueous solution without significant swelling or disintegration (Fig. S2C†). Interestingly, the response of human chondrocytes encapsulated within granular scaffolds fabricated with the GelMA or TEGDA crosslinkers was also equivalent over 4 weeks in culture (Fig. S3†). Chondrocytes remained similarly viable under both conditions, indicating a suitable microenvironment for cell survival (Fig. S3A†). Additionally, the mechanical properties of cartilage tissue are crucial for its functions in the body, especially the ability to sustain loads.^65^ The extent of scaffold stiffening was similar for the two sets of granular scaffolds (Fig. S3B†), enabled by the secretion of nascent extracellular matrix components from the encapsulated chondrocytes, including glycosaminoglycans (GAGs), collagen I, and collagen II (Fig. S3C†).

Similar *in vitro* chondrogenesis was therefore observed regardless of whether cell-binding motifs were present from the GelMA crosslinker. This indicates that the fully chemically-defined, synthetic system of granular zwitterionic hydrogels with the TEGDA crosslinker can substitute the hybrid system based on the GelMA crosslinker without impairing the cell–biomaterial interactions that enable chondrogenesis. Furthermore, since CBAA has a carboxylate pendant group, it would be amenable to bioconjugation chemistry in the future. Therefore, the hydrogels could be functionalized with amine-containing biomolecules to add in bioactive factors in a fully controlled manner. The scalable fabrication of this material platform along with the facile modulation of the scaffold properties offer great potential for expanding the versatility of zwitterionic microgel systems for tissue engineering.

### 3.2. Granular hydrogels with varied microgel stiffness and scaffold porosity

**Fig. 3:**
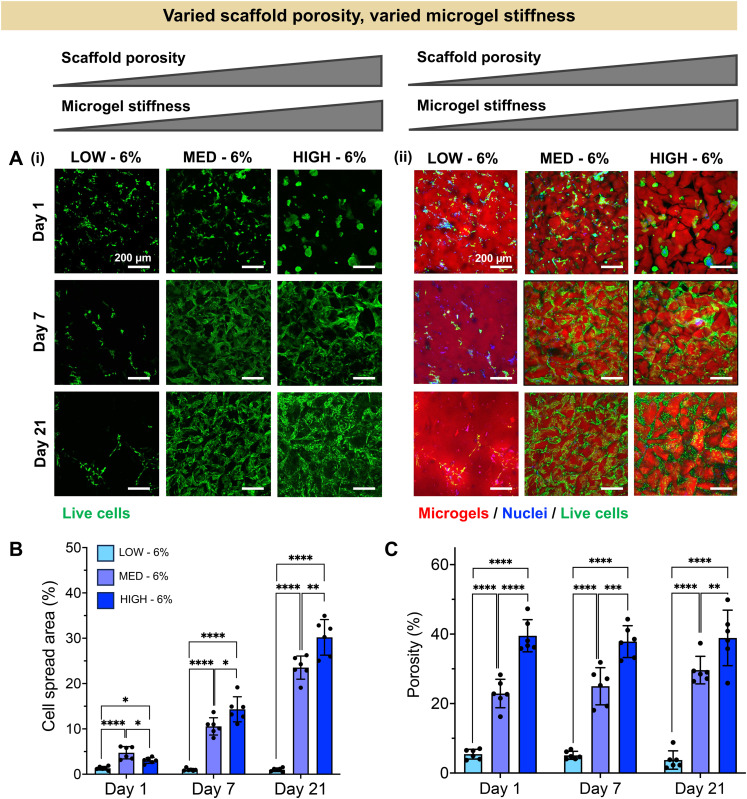
Human chondrocyte encapsulation in zwitterionic granular hydrogel scaffolds with constant wt% of microgels within the scaffold (6 wt%), creating granular hydrogels with varied scaffold porosity and varied microgel stiffness. (A) Representative images within LOW, MED, and HIGH–TEGDA crosslinking conditions indicating (i) the presence and spreading of living cells (Calcein AM, green), and (ii) the spatial relationship between living cells (Calcein AM, green) and microgels within the scaffold (rhodamine, red). (B) Quantification of cell spread area across the material conditions over time in culture. (C) Quantification of porosity across the material conditions over time in culture.

Having established a fully synthetic zwitterionic granular hydrogel platform and determined the effects of TEGDA crosslinking density on the scaffold properties, we next investigated the encapsulation of human chondrocytes within scaffolds with varied microgel stiffnesses and scaffold porosities (Fig. 3). Microgels were fabricated with LOW, MED, and HIGH TEGDA and resuspended at 6 wt% before the addition of cells. As described in Fig. 2, granular scaffolds with LOW TEGDA were made from bulk hydrogels of 800 Pa and had 5% porosity, granular scaffolds with MED TEGDA were made from bulk hydrogels of 2000 Pa and had 20% porosity, and granular scaffolds with HIGH TEGDA were made from bulk hydrogels of 3000 Pa and had 40% porosity. For cell encapsulation, human primary chondrocytes were obtained from corrective surgeries of young polydactyl patients.^53^ Since these cells are reported to be nonimmunogenic and immunosuppressive,^66^ they are a promising cell source for allogeneic cell-based therapies.

Stark differences were observed for the encapsulated cells between the different material conditions over 21 days in culture, as early as Day 1 (Fig. 3A). On the first day after cell encapsulation, the chondrocytes in LOW TEGDA scaffolds were round single cells without significant spreading, the chondrocytes in MED TEGDA scaffolds were single cells that had begun to spread along the microgels, and the chondrocytes in HIGH TEGDA scaffolds had aggregated into cell spheroids in the large void spaces between the microgels. Over time, the chondrocytes in LOW TEGDA scaffolds remained very sparse. On the other hand, the chondrocytes in MED and HIGH TEGDA scaffolds, despite having different cell morphologies on Day 1, were able to proliferate, spread, and form an interconnected network of cells around the microgels. The cell areas within MED and HIGH TEGDA scaffolds increased over time as proliferating cells filled in the initial void spaces. The cell area within the scaffolds on Day 1 was <5% for all conditions. On Day 21, the cell spread area was still <5% for LOW TEGDA scaffolds but was 25% and 30% for MED and HIGH TEGDA scaffolds, respectively (Fig. 3B). The porosities of the scaffolds were lowest for LOW TEGDA scaffolds and highest for HIGH TEGDA scaffolds at all timepoints, as the porosity for each cell-laden material condition did not change significantly over time (Fig. 3C, Fig. S3A†).

**Fig. 4:**
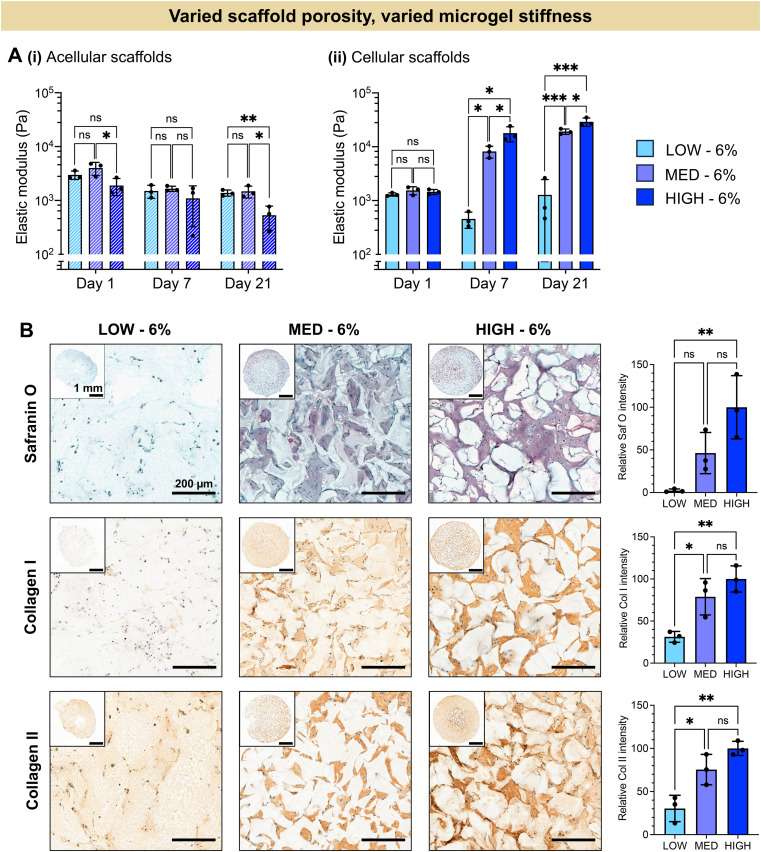
*In vitro* chondrogenesis of human chondrocytes encapsulated in zwitterionic granular hydrogel scaffolds with constant wt% of microgels within the scaffold (6 wt%), creating granular hydrogels with varied scaffold porosity and varied microgel stiffness. (A) Elastic modulus measured in compression of (i) acellular and (ii) cellular scaffolds during *in vitro* culture. (B) Representative histological staining and quantification for GAGs (Safranin O) and immunohistological staining and quantification for collagen I and collagen II.

The extent of chondrogenesis was analyzed within the scaffolds formed from LOW, MED, or HIGH TEGDA microgels, all resuspended at 6 wt% (Fig. 4). To restore function to damaged cartilage tissue, the implanted scaffold should provide a suitable microenvironment for the encapsulated chondrocytes to lay down nascent ECM, maturing the engineered tissue. To study tissue maturation, compressive modulus measurements were collected on Days 1, 7, and 21 (Fig. 4A). The initial moduli of scaffolds from LOW, MED, and HIGH TEGDA scaffolds are similar across material conditions, indicating that the stiffness of the overall scaffold is not significantly affected by the individual microgel stiffness from which the scaffold is comprised. For acellular control scaffolds, the modulus at each timepoint was similar across conditions and did not increase over time. The differences in stiffness over time observed for cellular scaffolds are therefore attributed to the effects of the encapsulated chondrocytes. On Day 1, all cellular scaffold conditions had an elastic modulus of 1 kPa. By Day 21, the LOW TEGDA scaffolds still had an elastic modulus of 1 kPa, while the MED and HIGH TEGDA scaffolds had elastic moduli of 20 kPa and 30 kPa, respectively. Therefore, greater engineered cartilage tissue maturation was observed for cellular scaffolds with greater amounts of TEGDA crosslinking.

The increased stiffness of the scaffold is attributed to the secretion of ECM from the chondrocytes. Histology and immunohistochemistry of the engineered tissue samples indicated the presence and localization of GAGs (stained red with Safranin O) and collagens (stained brown with immunohistochemistry) (Fig. 4B). GAGs and collagen II are major components of articular cartilage. The ECM is observed between the microgels of the scaffolds, with greater amounts of ECM for scaffolds with increasing TEGDA concentrations. Scaffolds with increased TEGDA crosslinker concentrations have both stiffer individual microgels and greater scaffold porosity, suggesting promising granular scaffold properties for cartilage tissue engineering.

It should be noted that we also observe collagen I deposition, which indicates tissue maturation toward fibrocartilage rather than hyaline cartilage. The hyaline cartilage found on the articulating surface of bones consists primary of type II collagen, whereas fibrocartilaginous tissues such as the meniscus also contain thicker, type I collagen fibers.^67,68^ It has been shown that native hyaline cartilage is more viscoelastic than fibrocartilage tissue.^69^ Moreover, scaffolds with more viscoelastic properties have been shown to enhance chondrogenesis towards hyaline cartilage.^70,71^ Future work could therefore include tuning the crosslinker mechanisms by adding more dynamic bonds to enhance the viscoelasticity of the scaffold in order to better mimic hyaline cartilage structure.

### 3.3. Granular hydrogels with varied microgel stiffness but constant scaffold porosity

**Fig. 5:**
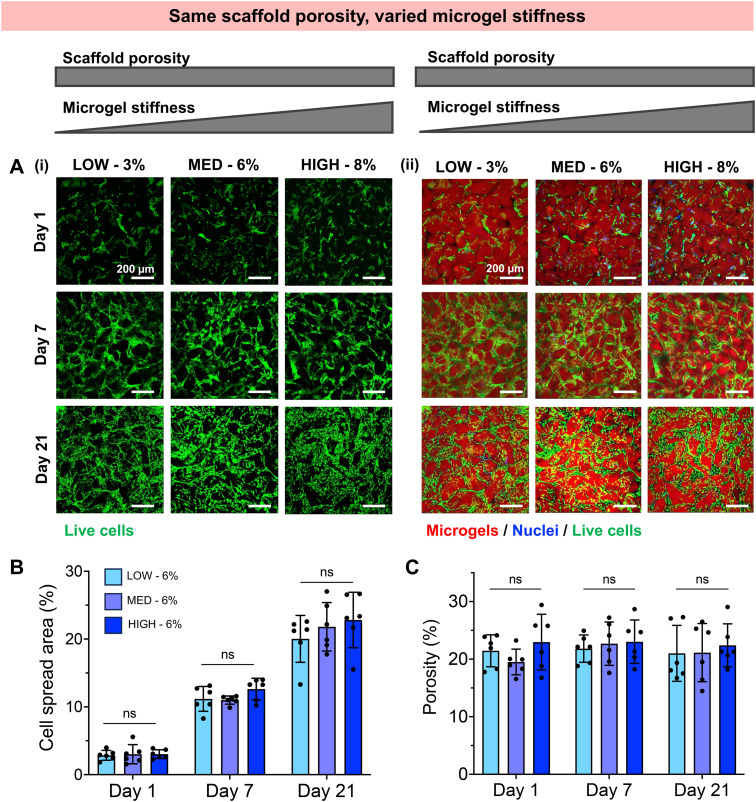
Human chondrocyte encapsulation in zwitterionic granular hydrogel scaffolds with constant scaffold porosity (∼20%) but varying microgel stiffness. (A) Representative images within LOW, MED, and HIGH–TEGDA crosslinking conditions indicating (i) the presence and spreading of living cells, and (ii) the spatial relationship between living cells (Calcein AM, green) and microgels within the scaffold (rhodamine, red). (B) Quantification of cell spread area across the material conditions over time in culture. (C) Quantification of porosity across the material conditions over time in culture.

We next investigated whether the different stiffness of the bulk gels from which the granular hydrogels were fabricated played a role in the extent of chondrogenesis observed, or whether chondrogenesis was dominated by the porosity of the scaffold. To do so, we studied granular scaffolds with varied degrees of crosslinking (*i.e.*, varied stiffness of the original bulk hydrogels) but the same scaffold porosity. To achieve the same scaffold porosity, LOW, MED, and HIGH TEGDA microgels were resuspended at 3, 6, and 8 wt% respectively before secondary crosslinking. Therefore, all three sets of scaffolds had 20% porosity. The granular scaffolds with LOW TEGDA were made from bulk hydrogels of 800 Pa, granular scaffolds with MED TEGDA were made from bulk hydrogels of 2000 Pa, and granular scaffolds with HIGH TEGDA were made from bulk hydrogels of 3000 Pa. Primary human chondrocytes were again encapsulated within the scaffolds for *in vitro* culture (Fig. 5).

**Fig. 6:**
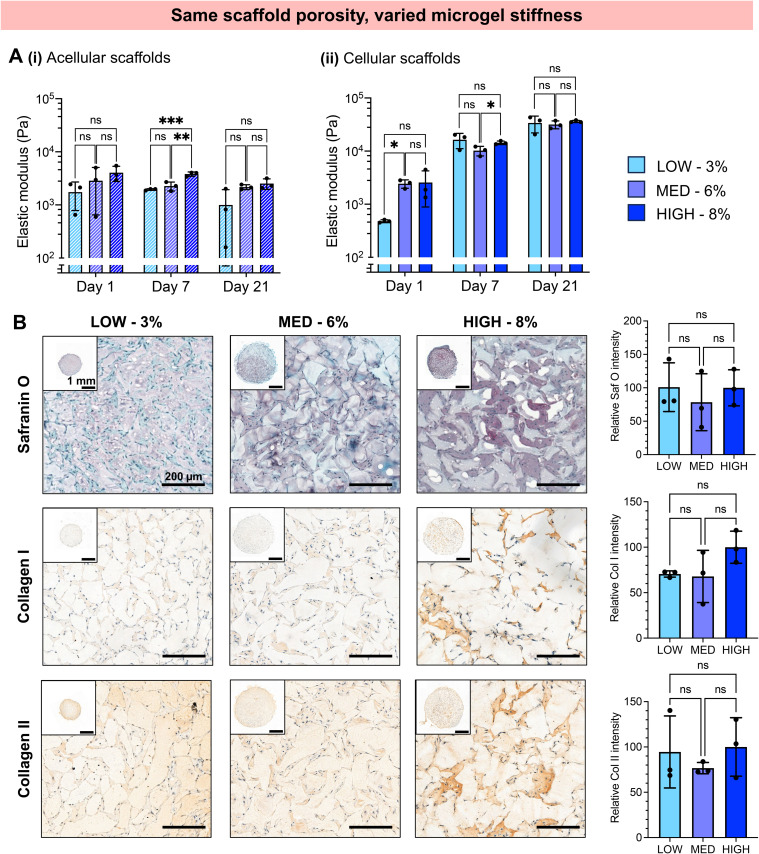
*In vitro* chondrogenesis of human chondrocytes encapsulated in zwitterionic granular hydrogel scaffolds with constant scaffold porosity (∼20%) but varying microgel stiffness. (A) Elastic modulus measured in compression of (i) acellular and (ii) cellular scaffolds during *in vitro* culture. (B) Representative histological staining and quantification for GAGs (Safranin O) and immunohistological staining and quantification for collagen I and collagen II.

Unlike the scaffolds that were matched by weight % but not porosity, these scaffolds with the same porosities guided encapsulated cells toward similar behaviors, despite differences in the stiffness of the individual microgels. For all conditions, chondrocytes started to spread on Day 1 and continued to proliferate and migrate over 21 days in culture to form interconnected cell networks between the microgels (Fig. 5A). The cell spread area increased for all conditions from <5% on Day 1, to 10% on Day 7, and to 20% on Day 21 (Fig. 5B). For these cell-laden scaffolds, the porosities remained constant at 20% porosity across all conditions and timepoints (Fig. 5C, Fig. S3B†). Similar indications of chondrogenesis and ECM secretion were also observed between granular scaffold conditions (Fig. 6). The compressive moduli of acellular scaffolds were <4 kPa for all timepoints and material conditions. On the other hand, while all cellular scaffolds were initially <3 kPa on Day 1, they all stiffened to ∼15 kPa on Day 7 and ∼35 kPa on Day 21 (Fig. 6A). Additionally, the secretion of GAGs and collagens as determined by histology and immunohistochemistry was similar across scaffold conditions at the end of the *in vitro* culture (Fig. 6B).

Since substantial differences were not observed for scaffolds with the same porosities but different microgel stiffnesses, the porosity of the scaffolds appeared to dominate over the stiffness of the constituent microgels. Scaffold porosity has been shown to significantly affect chondrogenesis of encapsulated cells, with increasing porosity favoring cell spreading and chondrogenic differentiation.^72–76^ Previous studies on granular hydrogel scaffolds for cartilage tissue engineering have commonly increased the porosity by increasing the microgel size, observing greater chondrocyte spreading and ECM production.^26,37,47,48^ In this study, we demonstrate that increasing the porosity of granular hydrogel scaffolds while keeping microgel size the same also results in enhanced chondrogenesis.

Previous works have shown that hydrogel stiffness can also affect chondrogenesis, and benefits of both soft, compliant 3D matrices and stiff matrices have been reported.^42–46,77,78^ Even small differences in hydrogel stiffness (0.5–1.5 kPa) have significantly affected the chondrogenic differentiation of encapsulated cells.^42^ However, in these previous studies, cells were encapsulated within bulk, nanoporous scaffolds. In our study, microgel stiffnesses within the range of 1–3 kPa did not have an evident effect on chondrocytes encapsulated in granular scaffolds with 20% void fractions. This might be due to the material stiffness not being sensed by cells as strongly in materials with such high porosities compared to a bulk hydrogel. Moreover, the pores are filled with nascent ECM over time in culture, further reducing cell–biomaterial interactions. In one study, it was shown that when chondrocytes are surrounded by a soft plasma clot, the hydrogel stiffness has no effect on the chondrogenesis.^43^ Our highly porous granular scaffolds may also promote cell–cell interactions over cell–biomaterial interactions, further reducing the effect of biomaterial mechanical properties on the cell phenotype.

To further validate this hypothesis, a greater range of microgel stiffnesses may be investigated in the future using our tunable biomaterial platform. Hydrogels with stiffnesses ranging between ∼200–200 000 Pa can be fabricated by further increasing or decreasing the concentration of the TEGDA crosslinker (Fig. S4†). Due to the wide range of possible microgel stiffnesses, which increases with decreasing EWC, a larger range of scaffold porosities may be studied while maintaining the same wt% of microgels in the scaffold. However, it should be noted that very stiff hydrogels might be challenging to (1) mechanically fragment into microgels and (2) stabilize after secondary crosslinking. Future work may explore the upper limit of scaffold porosity that enhances chondrogenesis while maintaining structural integrity and stability of the scaffold.

Based on our findings, we hypothesized that cells encapsulated in a microporous granular hydrogel sense the surrounding scaffold differently compared to a nanoporous scaffold, such that it may resemble more of a 2D environment in which cells are interacting with the surface of the microgels. We conducted a preliminary control experiment of chondrocytes seeded on bulk hydrogels with different stiffnesses (LOW, MED, HIGH TEGDA crosslinker) compared to those encapsulated in granular hydrogels with that same range of stiffnesses and similar porosities (Fig. S5†). However, after 24 h, when there was not yet much nascent ECM to interfere with cell–biomaterial interactions, cells still had a drastically different shape in 2D *vs*. within the pores of 3D granular scaffolds. While in 2D, cells were rounder and formed aggregates, but within the 3D microporous scaffold, cells adopted an elongated and spread morphology. Therefore, the microporous environment of the scaffold significantly contributes to the cell morphology and response as early as day 1 and is different from cells on a 2D hydrogel surface.

Finally, while the porosity of all cellular scaffolds remained constant over time in culture, we observed that acellular control scaffolds decreased in porosity over time (Fig. S6†). Since the TEGDA crosslinker includes ester groups, it is susceptible to hydrolytic cleavage. The decrease in acellular scaffold porosity may therefore indicate microgel swelling caused by degradation. This phenomenon was not observed for cellular scaffolds, indicating that the presence of cells and their nascent ECM may provide a physical barrier against microgel swelling behavior. The use of more readily degradable crosslinkers may be beneficial for long-term cultures, such that the microgels degrade and are replaced by nascent ECM over time.

## 4. Conclusions

Zwitterionic granular hydrogel scaffolds are uniquely well-suited for regenerative medicine applications, since the biocompatibility and low immunogenicity of zwitterionic hydrogels minimize adverse effects after implantation, and the microporosity of granular hydrogel scaffolds enhances cell behaviors associated with regeneration. We developed a zwitterionic granular hydrogel platform that allows for chemical and structural tunability to form a cell-instructive niche. By varying the degree of crosslinking of the zwitterionic hydrogels, we modulated the microgel stiffness and granular scaffold porosity both simultaneously and independently. We demonstrated the ability to match porosities within scaffolds comprised of microgels of different stiffnesses by adjusting the weight fraction of the microgels to account for their different equilibrium water contents. Leveraging this tunable material system elucidated how granular hydrogel properties affected chondrocyte behavior *in vitro*. The extent of chondrogenesis observed was dominated by the void fraction of the granular hydrogels compared to the stiffness of the constituent microgels, within the ranges tested here. This work helps inform the design of improved bioengineered therapies for cartilage tissue regeneration.

Furthermore, this study highlights the inherent difference in cell–biomaterial interactions between nanoporous *vs*. microporous scaffolds. Varying biophysical and biochemical properties of materials such as stiffness and ligand density has been shown to significantly affect cell response and chondrogenesis on nanoporous hydrogels.^79,80^ However, these characteristics might have a different effect and also to a different extent in a microporous 3D scaffold, as shown here for stiffness. Thus, as microporous granular scaffolds are becoming more widely used for tissue engineering applications, it is important to study such differences in this new platform. Moreover, cells in granular scaffolds are confined in the pores between microgels, as opposed to their homogenous distribution in a nanoporous scaffold. This phenomenon might result in differences in local cell densities for scaffolds having different overall porosities, which can also result in differences in cell–cell interactions. As the overall number of cells has a great influence on the extent of chondrogenesis and *in vitro* tissue maturation, we performed our study using the same overall cell densities. However, future experiments using adjusted cell densities that account for overall porosity could be conducted to better understand the effect of local cell densities on chondrogenesis.

Finally, since the material system developed in this work is fully synthetic and chemically defined, we expect that additional material engineering and tunability is possible for different applications and target tissues in future work. For example, peptides could be tethered into the zwitterionic hydrogels to introduce controlled concentrations and compositions of cell-adhesive ligands. Additionally, the small molecule crosslinker could be modulated with different degradation properties (*e.g.*, more or fewer hydrolytically cleavable moieties) to control the degradation rate of the scaffold while nascent ECM is secreted and remodeled by the encapsulated cells. Therefore, this work not only informs the design of enhanced granular hydrogel-based therapies for cartilage tissue regeneration but also lays the groundwork for future possibilities of tailored adaptations.

## Data availability

M. Asadikorayem, L. G. Brunel, P. Weber, S. C. Heilshorn, M. Zenobi-Wong, https://doi.org/10.3929/ethz-b-000658736, ETH Zürich Research Collection 2024.

## Conflicts of interest

The authors declare no conflict of interest.

## Supplementary Material

BM-012-D4BM00233D-s001
