# Supplementary material for: Porosity dominates over microgel stiffness for promoting chondrogenesis in zwitterionic granular hydrogels
Source: Biomater Sci. 2024 Sep 30;12(21):5504–20. doi: 10.1039/d4bm00233d (PMC11441418; doi:10.1039/d4bm00233d)
Supplement: BM-012-D4BM00233D-s001 [file BM-012-D4BM00233D-s001.pdf]

Supplementary Information (SI) for Biomaterials Science.
This journal is © The Royal Society of Chemistry 2024

Supporting Information (SI)

Porosity dominates over microgel stiffness for promoting chondrogenesis in zwitterionic granular hydrogels

Maryam Asadikorayem[1,†], Lucia G. Brunel[2,†], Patrick Weber[1], Sarah C. Heilshorn[3], Marcy Zenobi-Wong[1,*]

[1] Department of Health Sciences and Technology, ETH Zürich, Zürich, CH.

[2] Department of Chemical Engineering, Stanford University, Stanford, CA, USA.

[3] Department of Materials Science and Engineering, Stanford University, Stanford, CA, USA.

[†] M.A. and L.G.B. contributed equally to this work.

* Corresponding author: marcy.zenobi@hest.ethz.ch

Table S1. Chemical components of the zwitterionic bulk hydrogels crosslinked with TEGDA at LOW, MED, and HIGH concentrations.

**Chemical structure**	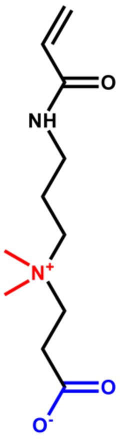	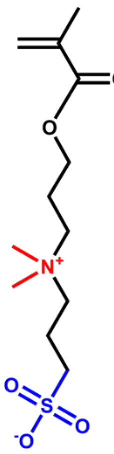	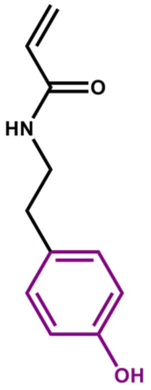	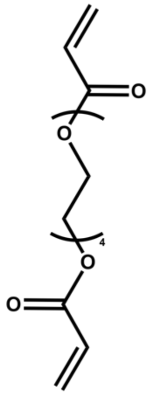
**Abbreviation**	**CBAA**	**SBMA**	**TyrAA**	**TEGDA**
	**Molarity (*mols per 1 mol of zwitterionic monomers*)**			
**LOW**	1.875 M (*0.75*)	0.625 M (*0.25*)	0.125 M (*0.05*)	**0.0025 M (*0.001*)**
**MED**	1.875 M (*0.75*)	0.625 M (*0.25*)	0.125 M (*0.05*)	**0.005 M (*0.002*)**
**HIGH**	1.875 M (*0.75*)	0.625 M (*0.25*)	0.125 M (*0.05*)	**0.0125 M (*0.005*)**

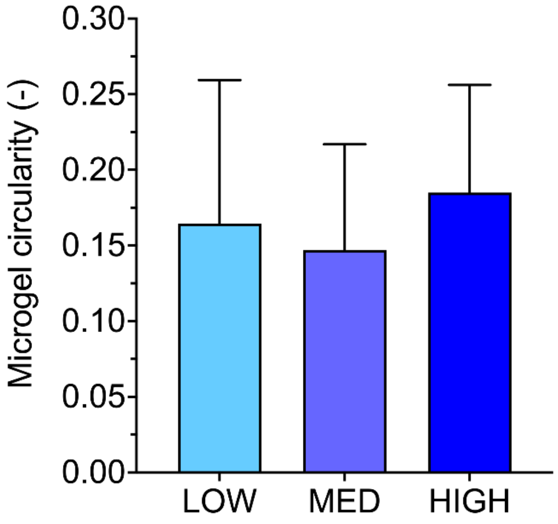

Figure S1. Microgels made with from bulk hydrogels with LOW, MED, and HIGH TEGDA concentrations have similarly low circularities, characteristic of the mechanical fragmentation technique by which they were fabricated. Circularity was calculated as 4π*Area/Perimeter^2, with a perfect circle having a circularity of 1.

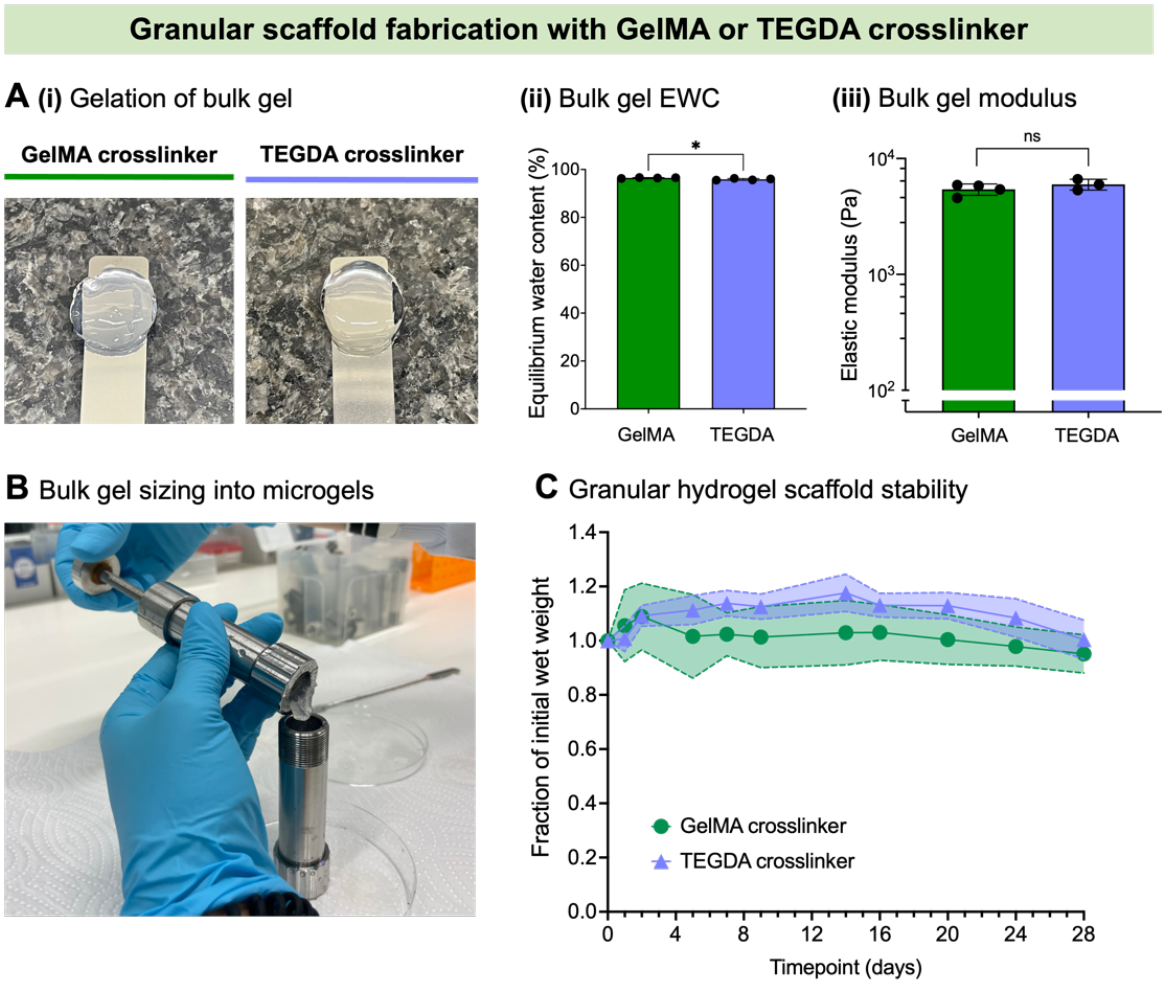

Figure S2. Fabrication of granular scaffolds from hydrogels crosslinked with GelMA or TEGDA. (A) GelMA and TEGDA crosslinkers enable similar **(i)** gelation of a zwitterionic bulk hydrogel, **(ii)** equilibrium water content, and **(iii)** elastic modulus measured in compression. **(B)** The bulk hydrogels are sized by mechanical fragmentation into microgels through repeated manual extrusion through a sieve. **(C)** The wet weights of granular hydrogel scaffolds are stable over 28 days of incubation, for scaffolds fabricated from hydrogels crosslinked with either GelMA or TEGDA. The solid lines and shaded regions represent the means and standard deviations, respectively.

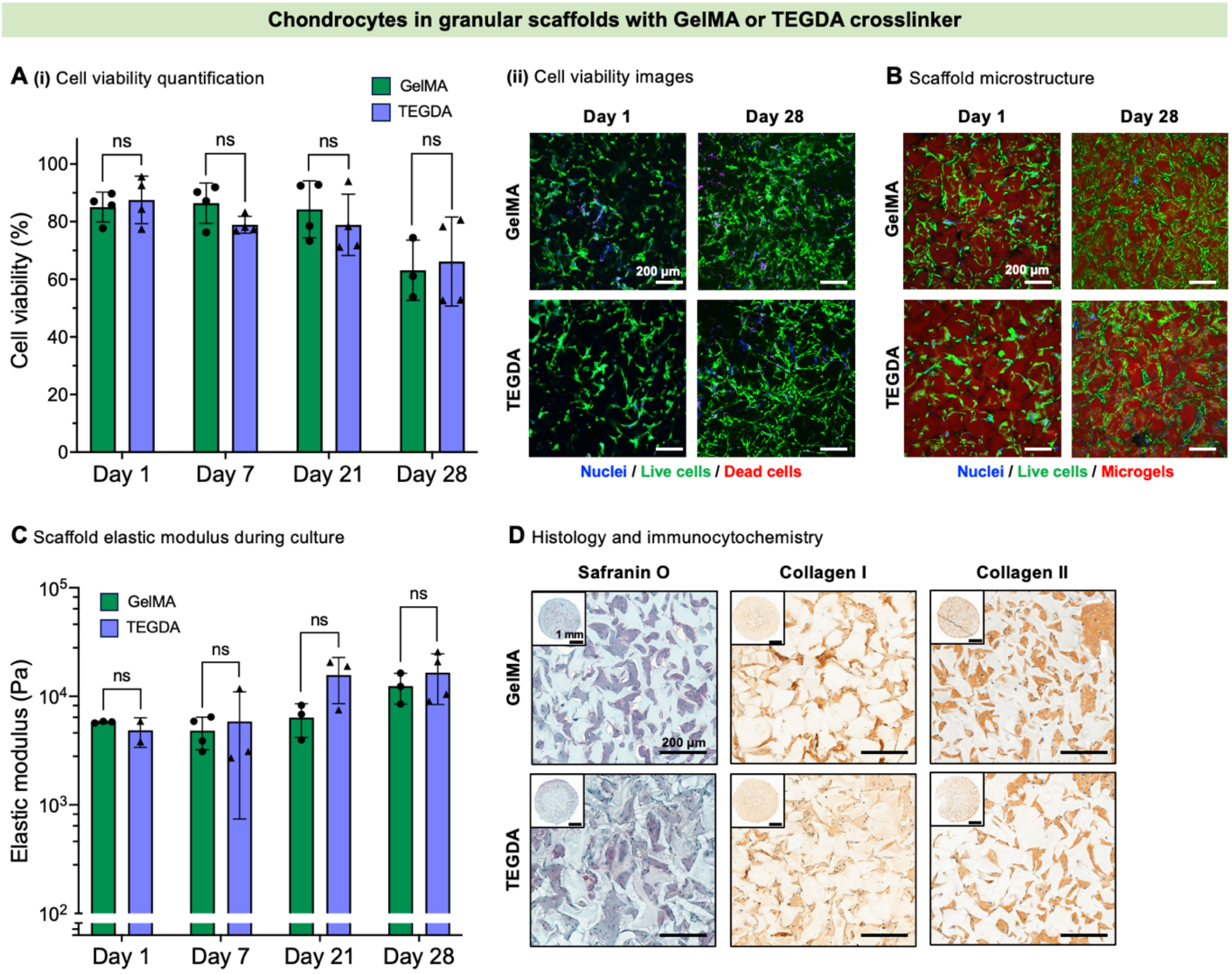

Figure S3. Human chondrocyte encapsulation and chondrogenesis within zwitterionic granular hydrogel scaffolds fabricated with the GelMA or TEGDA crosslinker. (A) (i) Quantification of the chondrocyte viability from a Live/Dead assay. **(ii)** Representative images of the Live/Dead assay, showing the cell nuclei (Hoechst, blue), live cells (Calcein AM, green), and dead cells (PI, red). **(B)** Representative images of living cells (Calcein AM, green) in the fluorescently-labeled granular scaffold (rhodamine, red). **(C)** Elastic modulus measured in compression of cellular scaffolds during *in vitro* culture. **(D)** Representative histological staining for GAGs (Safranin O) and immunohistological staining for collagen I and collagen II.

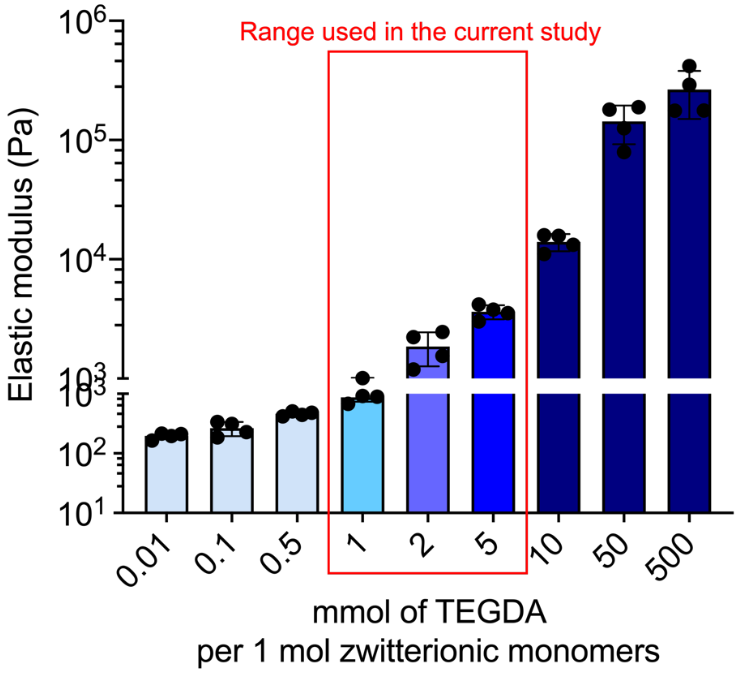

Figure S4. Bulk hydrogels with a wide range of stiffness can be achieved by varying the concentration of crosslinker. Bulk hydrogels were made with 0.01 - 500 mmol of TEGDA crosslinker per 1 mol of zwitterionic monomers, resulting in gels with stiffnesses ranging from ~ 200 - 200,000 Pa.

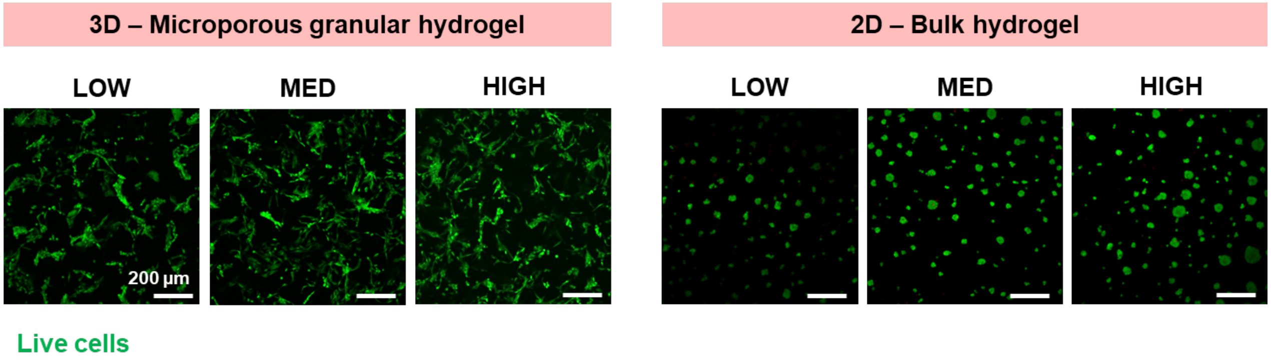

Figure S5. (Left) Chondrocytes encapsulated in 3D granular hydrogel scaffolds with different microgel stiffnesses (LOW, MED, HIGH) and similar porosities *vs*. (Right) chondrocytes seeded in 2D on top of bulk hydrogels with the same range of stiffnesses. The cell morphology was drastically different for cells encapsulated in 3D microporous granular hydrogels compared to cells seeded on 2D bulk hydrogels, suggesting that the observed cell-biomaterial interaction in granular scaffolds is not only a 2D interaction with the microgels. For the 2D cell experiment, bulk hydrogels were made by photocrosslinking of sterile filtered precursor solution as described in methods section 2.3.1, and chondrocytes at passage 3 were seeded as 30,000 cells/cm^2 and cultured with chondrogenic media.

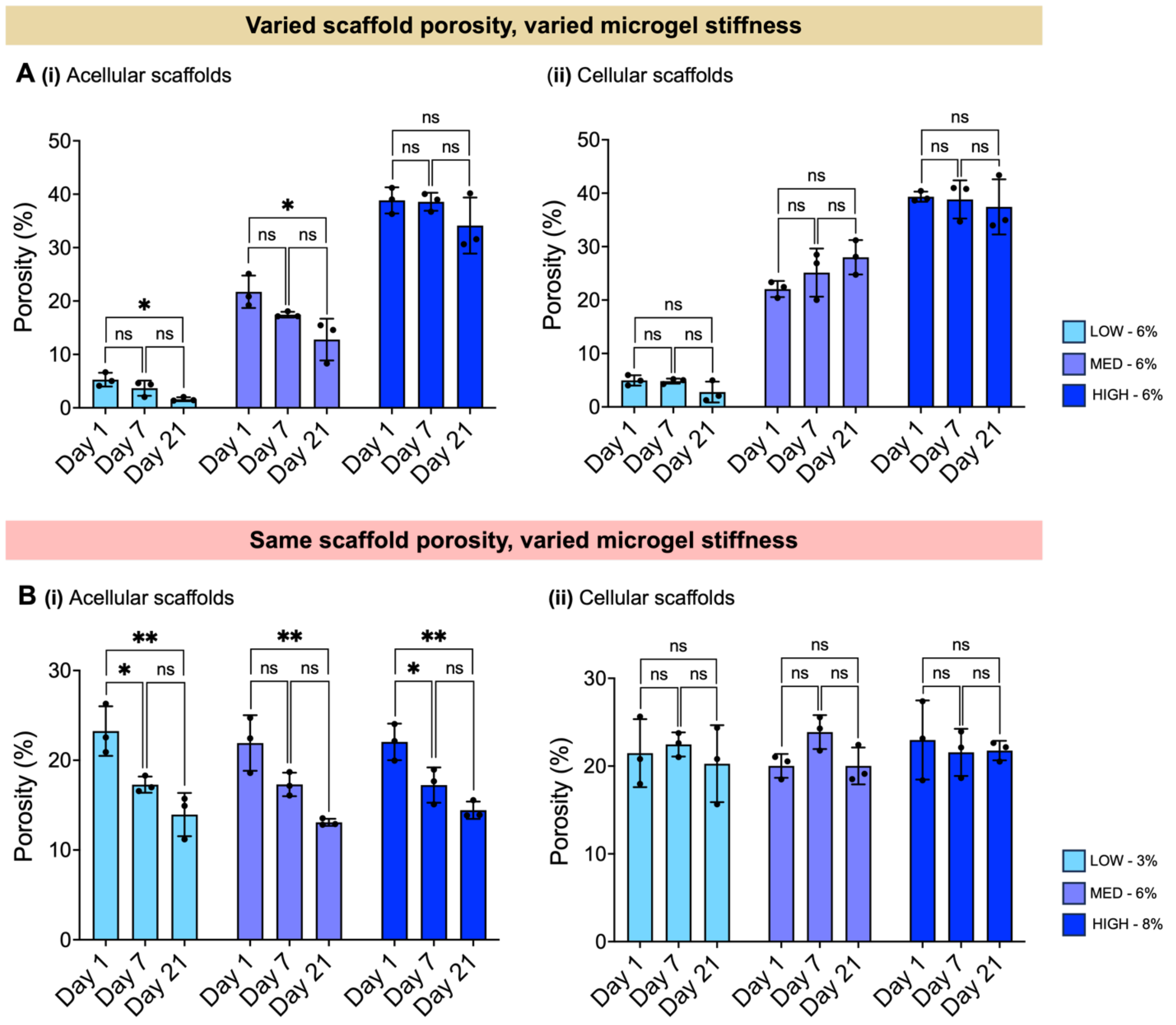

Figure S6. Porosity of granular hydrogel scaffolds over time in incubation. (A) Porosity of **(i)** acellular and **(ii)** cellular scaffolds for LOW, MED, and HIGH-TEGDA crosslinking conditions, in which all microgels were resuspended at 6 wt% for the granular scaffolds. **(B)** Porosity of **(i)** acellular and **(ii)** cellular scaffolds for LOW, MED, and HIGH-TEGDA crosslinking conditions, in which microgels were resuspended at 3 wt% for LOW-TEGDA, 6 wt% for MED-TEGDA, and 8 wt% for HIGH-TEGDA for the granular scaffolds, such that the initial scaffold porosity was matched between conditions.
